# Controlling quantum interference in phase space with amplitude

**DOI:** 10.1038/s41598-017-02540-3

**Published:** 2017-05-23

**Authors:** Yinghong Xue, Tingyu Li, Katsuyuki Kasai, Yoshiko Okada-Shudo, Masayoshi Watanabe, Yun Zhang

**Affiliations:** 1 Department of Engineering Science, The University of Electro-Communications, 1-5-1 Chofugaoka, Chofu-shi Tokyo, 182-8585 Japan; 2 Department of Physics, East China University of Science and Technology, Meilong Road 130, Shanghai, 200237 China; 3 College of Information Engineering, Taiyuan University of Technology, Taiyuan, 030024 Shanxi China; 4 Advanced ICT Research Institute, National Institute of Information and Communications Technology, 588-2, Iwaoka, Nishi-ku, Kobe, Hyogo, 651-2492 Japan

## Abstract

We experimentally show a quantum interference in phase space by interrogating photon number probabilities (n = 2, 3, and 4) of a displaced squeezed state, which is generated by an optical parametric amplifier and whose displacement is controlled by amplitude of injected coherent light. It is found that the probabilities exhibit oscillations of interference effect depending upon the amplitude of the controlling light field. This phenomenon is attributed to quantum interference in phase space and indicates the capability of controlling quantum interference using amplitude. This remarkably contrasts with the oscillations of interference effects being usually controlled by relative phase in classical optics.

## Introduction

In quantum optics, the optical phase space is a useful tool in which all quantum states of an optical system can be represented. It originally provides a bridge between classical and quantum physics and usually consists of two quadrature amplitudes, which correspond to the real and imaginary parts for complex amplitude of an optical field^1^. A plot of the quadrature against each other, which is called phase diagram, can give insight into the properties and behaviors of the system that might otherwise not be obvious, such as the nonpositive character of Wigner function for a Fock state^2, 3^. Furthermore, the Wigner function has also contributed to manifestation of quantum nonlocality and nonclassicality of field^4, 5^. The quantum interference in phase space is an essential concept and it gives a convincing interpretation for many quantum phenomena in modern physics^6^. For example, it has been successfully applied to directly interpret the squeezed vacuum state with an even number distribution and to predict the squeezed state with an oscillation photon number^7, 8^. Unfortunately, it is still a challenging task to directly demonstrate this interference in experiment. Up to now, most of experimental achievements were mainly focused on the presentations of different quantum state in phase space, such as squeezed state, single-photon state, Schödinger cat state and so on^9–13^. And the photon number distribution of a quantum state is usually obtained by reconstructation of state density matrix using the well-developed quantum-state tomography^14–16^. Although, most of features of quantum interference in phase space were discovered in recent years, it still remains many mysteries^17, 18^. The purpose of this paper is to directly demonstrate the quantum interference in phase space and to discover its novel features in experiment. We found that oscillations of interference in phase space can be controlled by amplitude of field, contrasting to that these oscillations are usually controlled by relative phase both in classical and quantum physics. The observation of this phenomenon was also proposed by interfering two arbitrary number states on a beam splitter with variable transmission and reflection coefficients^19^. Curiously, this oscillation can be understood the particle nature of the quantum system becasue the phase space consists of information of two quadrature amplitudes for a photon state. In physics, these two quardature are corresponding position and momnentum of a virtual particle respectively.

On the other hand, optical parametric amplification of a coherent state is a most effective test-bed for demonstrating the two-photon interference, which acts as an essential role in many applications of quantum information and quantum communication protocols with discrete variable. The experimental demonstration of two-photon interference effect has been performed with both continuous-wave and pulsed laser sources^20, 21^. Meanwhile, this phenomenon was also observed by interfering a two-photon state from spontaneous parametric down conversion (SPDC) and a weak coherent state on a beam splitter^22, 23^. More recently, the reconstruction of the full complex wave function (temporal or spectral) of two-photon probabilty amplitude has also been completed^24–26^. As an application, generation of single-photon sate for a long distance quantum key distribution was proposed when destructive two-photon interference occurs^27, 28^. Furthermore, the concept of two-photon interference was extended to multi-photon interference and proposed to produce a high-NOON state, which is the most important light source for enhancing phase measurement sensitivity to Heisenberg limit^29, 30^. To date, most of existing experimental realizations of such states have based on interference of quantum states or state projection to the NOON component of initial N-photon states^31, 32^. As a matter of fact, the generation of N-photon sates is principally based on the quantum interference of two-photon state from SPDC and weak coherent state. So, further improvements of the performance of such a system rely on the investigation of quantum interference. Especially, it is more important to find new control parameters of the quantum interference. Here, we experimentally demonstrate the quantum interference in phase space using a pulsed optical parametric amplifier (OPA). By recording two-fold, three-fold or four-fold coincidence of the field, the obtaining two-photon, three-photon or four-photon probability was studied. Remarkably, we have observed that the probabilities exhibit oscillations depending on the amplitude of injected control light. This may open another path for understanding the quantum phenomena in phase space and provide another novel resource for quantum information and quantum measurement technology.

## Model

**Figure 1:**
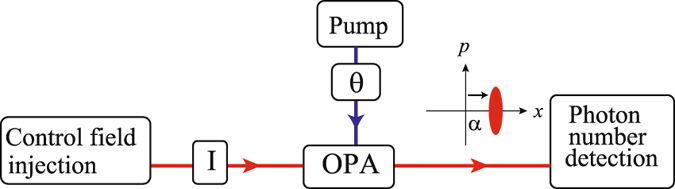
Sketch of the generation and detection of DSS.

**Figure 2:**
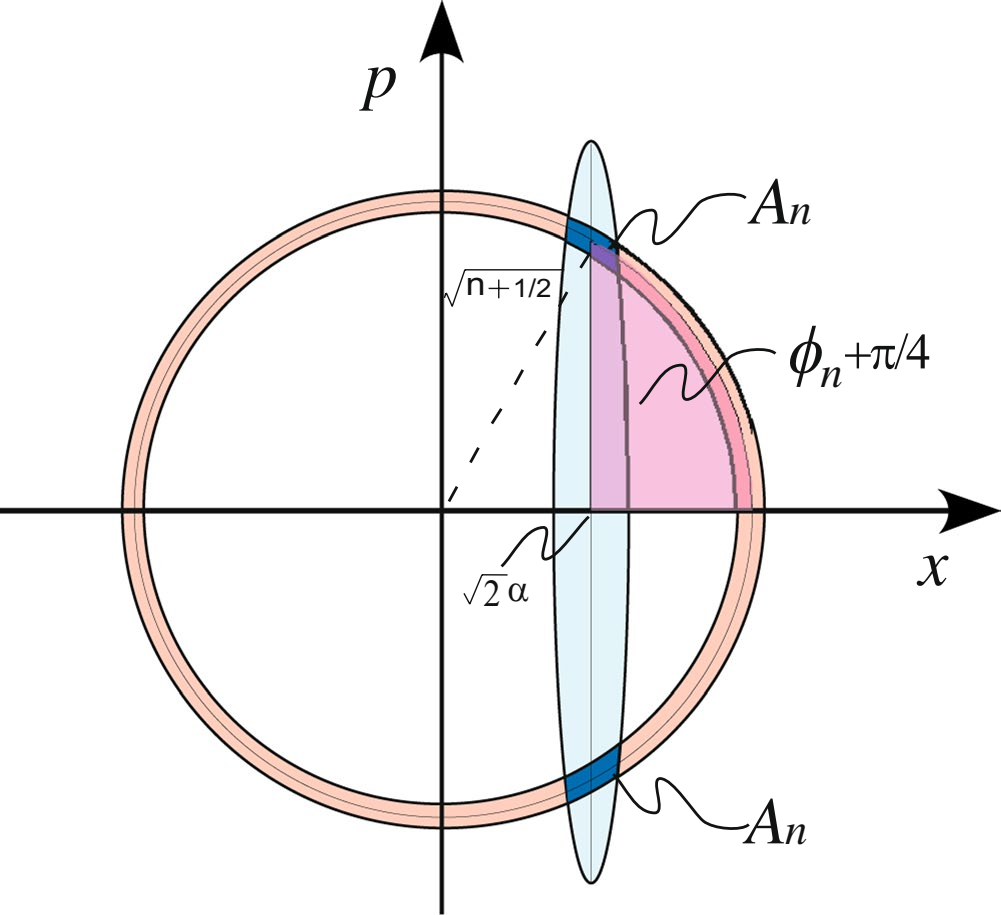
A simple mode of a displaced squeezed state and a n-photon number state in phase space. The circular band represents the n-photon number state. The ellipse represents the DSS. The overlaps correspond to two interfering complex-valued probability amplitudes. The phase for one probability amplitude is fixed by the pink shaded domain caught by the central lines of two states.

The demonstration was implemented by a displaced squeezed state (DSS), which is generated by displacing a squeezed vacuum state in the phase space. In experiment, it was realized by injecting a coherent field as a control light into the parametric down converter as shown in Fig. 1. The displacement can be controlled by adjusting power of the control light. Such a system is exactly the same as the previous reports, in which two photon interference effects were investigated^20, 21^. But we present the argument of the particle behavior for the output field in the phase space. To understand more rigorously how this happens, let us represent the output field from OPA by the phase space distribution

1 $${W}_{sq}(x,p)=\frac{2}{\pi }\,\exp \,[-{e}^{-2r}{(x-\sqrt{2}\alpha )}^{2}]\,\exp \,[-{e}^{2r}{p}^{2}],$$

where *r* is squeezing parameter, which is relative to pump power in experiment, and *α* is the displacement. Without loss the generality, we consider the displacement on the direction of *x*, *i.e. α* is a positive real number. In phase space, finding n-photon number probability is determined by two probability amplitudes, which corresponds to two overlaps between n-th band for n-number state and Gaussian cigar for DSS. A simple explanation for probability amplitude (*A*_*n*_) and their phase (*ϕ*_*n*_) is shown in Fig. 2. They have the same magnitudes *A*_*n*_ and opposite phases *ϕ*_*n*_^7^,

2 $${A}_{n}=\mathop{\underbrace{\int dx\int dp}}\limits_{{\rm{n}} \mbox{-} {\rm{th}}\,{\rm{photonband}}}\,{W}_{sq}(x,p),$$

3 $${\phi }_{n}=(n+\frac{1}{2})\,\arctan \,[\frac{1}{\alpha }\sqrt{n+\frac{1}{2}-{\alpha }^{2}}]-\alpha \sqrt{n+\frac{1}{2}-{\alpha }^{2}}-\frac{\pi }{4}.$$

According to the concept of interference in phase space, the probability for finding n-photon number is

4 $${W}_{n}=4{A}_{n}{\cos }^{2}{\phi }_{n}\mathrm{.}$$

**Figure 3:**
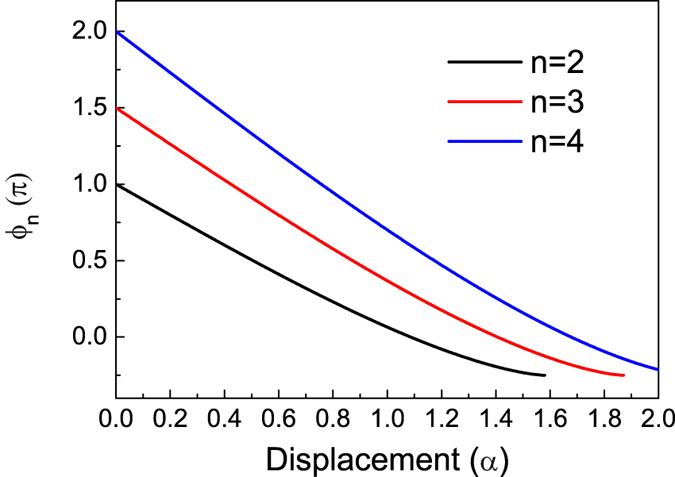
Phase associated with overlap in phase space (*ϕ*_*n*_) as a function of displacement (*α*) for n = 2, 3, and 4.

We note that *ϕ*_*n*_ is governed by the displacement (*α*) of DSS for a fixed n. In this sense, the oscillation of the probability for finding n-photon number state can be controlled by the amplitude of the control light. In equation (3), it indicates that the phase changes from *nπ*/2 to − *π*/4 with the increase of displacement. Thus, *W*_*n*_ starts from its maximum value of 4*A*_*n*_ and oscillates with the increasing displacement when *n* is even. Meanwhile, it starts from its minimum value of 0 and also oscillates with the increasing displacement when *n* is odd. Figure 3 gives *ϕ*_*n*_ as a function of displacement (*α*) for n = 2, 3, and 4. It clearly shows that the *ϕ*_*n*_ almost linearly depends on the displacement. Hence, we can obtain *n*/2 periodic oscillation for even *n* and (*n* − 1)/2 periodic oscillation for odd *n*, respectively.

## Methods

**Figure 4:**
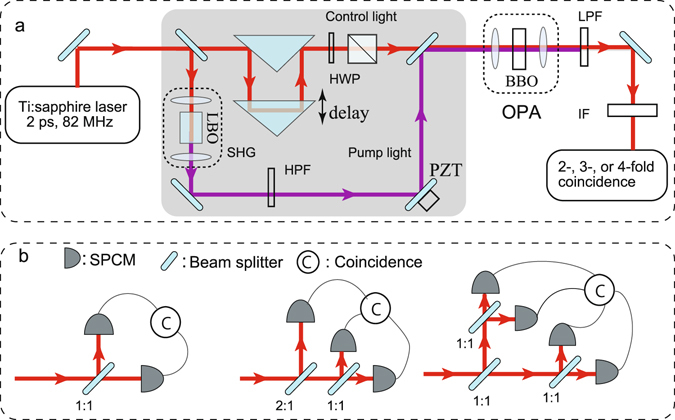
Experimental setup for quantum interference in phase space. (**a**) Layout of the setup; (**b**) Detection systems.

The layout of the setup is illustrated in Fig. 4 (a). The primary source of the experiment is a cw mode-locked Ti:Sapphire laser, operating at 798 nm with a pulse duration of 2 ps and a pulse repetition rate of 82 MHz. Most part of laser powers was sent to a single pass through second harmonic generator (SHG) to produce an efficient 399 nm light for pumping the OPA. In our system, a 15-mm-long LBO crystal is used as a nonlinear material. In particular, an average power of about 100 mW of ultraviolet light was generated when the fundamental input power is about 700 mW. However, in the following experiments, about 30 mW of ultraviolet light is employed to pump the OPA. This power is strong enough to obtain a significant single-pass parametric gain in our OPA. The smaller remainder portion of the coherent laser as the control light was combined with pump light and injected into the OPA for producing DSS and controlling the displacement. The OPA consists of a 3-mm-long type I beta barium borate (BBO) crystal. The photon number probability of generated DSS was investigated with two- three- or four fold coincidence counter, which consists of beam splitter, fiber coupled single photon counting module (SPCM, Perkin Elmer, SPCM-AQRH14), electronical coincidence circuit, and a photon counter. The detection systems are shown in Fig. 4(b). The high pass filter (HPF) in the pump arm was used to cut the residual fundamental light of SHG. The displacement is varied by changing the power of control light. To do it, a variable attenuator, which consists of a half-wave plate (HWP) and a polarizing beam-splitter (PBS) is inserted into the arm of control light. Interference filters (IF) and low pass filter (LPF) are placed before each fiber coupler in order to avoid dark counts from room scattering light and the residual pump light, the bandwidth of IF is 15 nm.

To ensure the control light simultaneous arriving OPA with pump pulses, a pair of movable prisms were employed for temporally delaying it. The relative phase between the control light and the pump light was also finely adjusted and stabilized by a mirror, which is mounted on a piezoelectric translator (PZT). The observability in this experimennt is crucially dependent on the stability of the relative phase between the control light and the pump. In order to improve the stability, the two beams are aligned as collinearly as possible. Furthermore, all optical components for SHG and control light were arranged on one breadboard. Thanks to the stability of the whole system, OPA was able to stably operate at de-amplification more than 10 minutes only by employing DC voltage on the PZT without any further active feedback control. Thus, the system was used to produce an amplitude DSS in phase space. For one measurment, the relative phase was adjusted to keep at de-amplification and was monitored by detecting part of the control light during the measurment. The displacement was varied by changing the power of the control light. To realize this control, a variable attenuator, which consists of a half-wave plate (HWP) and a polarizing beam-splitter (PBS) was inserted in the arm of the control light. The effective squeezing parameter *r* was estimated to be 0.15 from the measured parametric gain with different pump powers by taking into account the mode matching efficiency between the pump light and the control light^33, 34^. A minimum deamplification of −0.6 dB and maximum amplification of 0.8 dB were obtained at the provided pump power of about 30 mW. This corresponds to an average photon number of 0.02. The photon number probability of the generated DSS was investigated with our detection systems. The observed single count rates of the produced squeezed vacuum sate were 10 kcps and its two-fold coincidence count rate was around 3 kcps without injection of the control light. Hence, the dark counts of 100 cps for SPCM can be neglected and a quantum detection efficiency of 25% was estimated^35^.

## Results and Discussion

**Figure 5:**
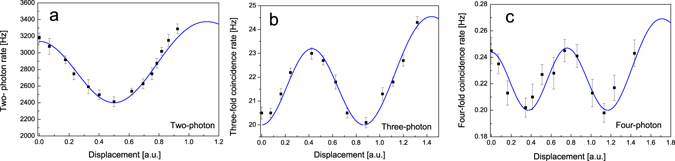
Experimental results: Coincidence measurements demonstrating obtaining N-photon probability for N = 2, 3 and 4 with no background subtraction. Error bar indicate standard deviation for five measurements. Solid curves are obtained using analytical model.

Figure 5(a) shows the two-fold coincidence count rate with various input control light powers, where all the powers are normalized to displacement (*α*). They are also checked from the counting rates when the input power of the control light was varied without the pump light. Solid curves are obtained using analytical model in equation (4). We note that both probability amplitude (*A*_*n*_) and their phase (*ϕ*_*n*_) are able to approximate to a linear function as displacement (*α*) in our case. Hence, a simple analytial model of ${W}_{n}=({C}_{1}+{C}_{2}\alpha ){\cos }^{{\rm{2}}}(n\pi /2-{C}_{3}\alpha )$, where *C*_1_ is the measured coincidence rate at *α* = 0 and *C*_*i*_(*i* = 2,3) were relative to photon number *n* and squeezing parameter *r*, was used to fit experimental data. The two-fold coincidence corresponds to the obtained two-photon number state probability of DSS when contributions for higher photon states (more than two-photon state) are negligible. This is always true for such kind of experiment, since the average photon numbers of both the squeezed vacuum state and the weak coherent state are small and the higher-photon events are negligible comparing with the two-photon event. We can see that the two-fold coincident has its maximum value without injection of the control light (corresponding to *φ* = *π*). As the displacement increases, it is decreasing and reaches its minimum at an optimum displacement (corresponding to *φ* = *π*/2). For further increase of displacement, the measured two-fold coincidence monotonously rises, because the higher-photon events from the control light become unignorable. Three-fold and four-fold coincidences, which correspond to the probabilities of obtaining three-photon and four-photon states, as a function of displacement (*α*) are shown in Fig. 5(b) and (c), respectively. As mentioned above, the three-fold coincidence indicates two minimum values (corresponding to *φ* = 3*π*/2, *π*/2) and one maximum values (corresponding to *φ* = *π*). Meanwhile, the four-fold coincidence gives two maximum values (corresponding to *φ* = 2*π*, *π*) and two minimum values (corresponding to *φ* = 3*π*/2, *π*/2). Because the higher photon events become significant with the further increase of displacement, all the maximum values of *φ* = 0 are not observed.

In the experimental results, all the three measured coincidences clearly show that the probabilities of obtaining the n-photon number states have oscillating characteristics with the increase of displacement (*α*). They can be analyzed with the above-mentioned analytical model by introducing a parameter of visibility. The solid curve is *A*_*n*_[1 + *V*(1 + *C*_*n*_ *α*) cos(2*φ*_*n*_)] where V is the expected visibility, *A*_*n*_ and *C*_*n*_ come from a fit to the data. It indicates that the visibility of 27%, 17%, and 19% for the two-photon, three-photon, and four-photon states, respectively, without taking into account of dark noise. The low visibility may be limited by bandwidth of interference filter and mode matching efficiency between the pump light and the control light. Further improvements are possible by employing a narrow bandwidth filter and a waveguide as nonlinear material. It is clearly shown that the two-fold and three-fold coincidences have single periodic oscillation characteristics and the four-fold coincidence has doubly periodic oscillation characteristics. These results well agree with the expectation from the above-mentioned concept of quantum interference in phase space. In contrast to the previous experimental investigations, in which the photon statistics were obtained by the technique of quantum tomography, our observation is implemented by observing the multi-photon interference^8, 14^. It provides a direct demonstration of the concept of quantum interference in phase space. On the other hand, quantum tomography technique was usually employed to measure quantum state, the present results show a potential method to prepare quantum state, such as high-photon Fock state and high-NOON state, by the principle of quantum interference in phase space.

We can also visualize that an elongated ellipse representing squeezed state moves along the real part axis in phase space when the displacement is increased. The probability to find n photon in the state is hence given by superposition of two probabilities amplitudes, which are two overlaps between ellipse and a circular band representing *n* photon number sate in space. The phase difference is the area enclosed by the envelope of two states. The displacement changes the phase difference and interference oscillation occurs. Thus, the oscillations can be controlled by amplitude.

It is interesting that the control of quantum interference with amplitude gives another novel feature of particle interference. The output field of OPA can be considered as a combination of the squeezed vacuum state and the weak coherent state (control light). In the case of two-photon probability, it has been well understood by the concept of two-photon interference. It is, however, not easy to understand how the three-photon probability oscillates with the amplitude of the control light because only even photon number states probability amplitudes exist in squeezed vacuum state. What happens is that a two-photon event from squeezed vacuum state combines with a single-photon event in the control light to form a three-photon probability amplitude. This probability amplitude interferes with another three-photon probability amplitude from the control light. Thus, the amplitude of the control light governs the three-photon event in the DSS. For the four-photon state, the interference includes the four-photon probability amplitudes from the control light and the squeezed vacuum state, and the combined four-photon probability amplitude formed by two-photon events from the control light and the squeezed vacuum state, respectively. Thus, the four-photon event is also governed by the amplitude of control light.

In conclusion, we directly demonstrated quantum interference in phase space. The amplitude control of quantum interference was experimentally discovered as another novel feature of particle interference. The extending concept of quantum interference in phase space is extremely fruitful. These results represent another step toward the quantum state engineering and may provide new resources for quantum information and measurement protocols.
